# Was Frozen Mammoth or Giant Ground Sloth Served for Dinner at The Explorers Club?

**DOI:** 10.1371/journal.pone.0146825

**Published:** 2016-02-03

**Authors:** Jessica R. Glass, Matt Davis, Timothy J. Walsh, Eric J. Sargis, Adalgisa Caccone

**Affiliations:** 1 Department of Ecology and Evolutionary Biology, Yale University, New Haven, Connecticut, United States of America; 2 Division of Vertebrate Zoology, Yale Peabody Museum of Natural History, Yale University, New Haven, Connecticut, United States of America; 3 Department of Geology and Geophysics, Yale University, New Haven, Connecticut, United States of America; 4 Bruce Museum, Greenwich, Connecticut, United States of America; 5 Department of Anthropology, Yale University, New Haven, Connecticut, United States of America; 6 Institute for Biospheric Studies, Molecular Systematics and Conservation Genetics Center, Yale University, New Haven, Connecticut, United States of America; Perot Museum of Nature and Science, UNITED STATES

## Abstract

Accounts of woolly mammoths (*Mammuthus primigenius*) preserved so well in ice that their meat is still edible have a long history of intriguing the public and influencing paleontological thought on Quaternary extinctions and climate, with some scientists resorting to catastrophism to explain the instantaneous freezing necessary to preserve edible meat. Famously, members of The Explorers Club purportedly dined on frozen mammoth from Alaska, USA, in 1951. This event, well received by the press and general public, became an enduring legend for the Club and popularized the notorious annual tradition of serving rare and exotic food at Club dinners that continues to this day. The Yale Peabody Museum holds a sample of meat preserved from the 1951 meal, interestingly labeled as a South American giant ground sloth (*Megatherium*), not mammoth. We sequenced a fragment of the mitochondrial cytochrome-b gene and studied archival material to verify its identity, which if genuine, would extend the range of *Megatherium* over 600% and alter our views on ground sloth evolution. Our results indicate that the meat was not mammoth or *Megatherium* but green sea turtle (*Chelonia mydas*). The prehistoric dinner was likely an elaborate publicity stunt. Our study emphasizes the value of museums collecting and curating voucher specimens, particularly those used for evidence of extraordinary claims.

## Introduction

One of the first scientific accounts of a well-preserved woolly mammoth (*Mammuthus primigenius*) frozen in Siberia described the meat as enticingly red and marbled but smelling so putrid that researchers could only tolerate a minute in its proximity [1]. Despite this initial review, numerous apocryphal tales exist of dinners made from centuries-old mammoths found frozen whole in clear blocks of ice [2,3]. These accounts have not only enchanted the public but also heavily influenced early scientific thought on Quaternary extinctions and climate; many resorting to catastrophism to explain the instantaneous freezing necessary to preserve palatable meat [3]. The possibility of cloning is now the major draw of frozen mammoths [4] but the public remains curious about eating prehistoric meat [5], especially because some modern paleontologists have credibly described tasting mammoth and extinct bison found preserved in permafrost [6,7]. Although less publicized today, eating study specimens was once common practice for researchers [8]. Charles Darwin belonged to a club dedicated to tasting exotic meats [9], and in his first book wrote almost three times as much about dishes like armadillo and tortoise urine than he did on the biogeography of his Galapagos finches [10].

**Fig 1:**
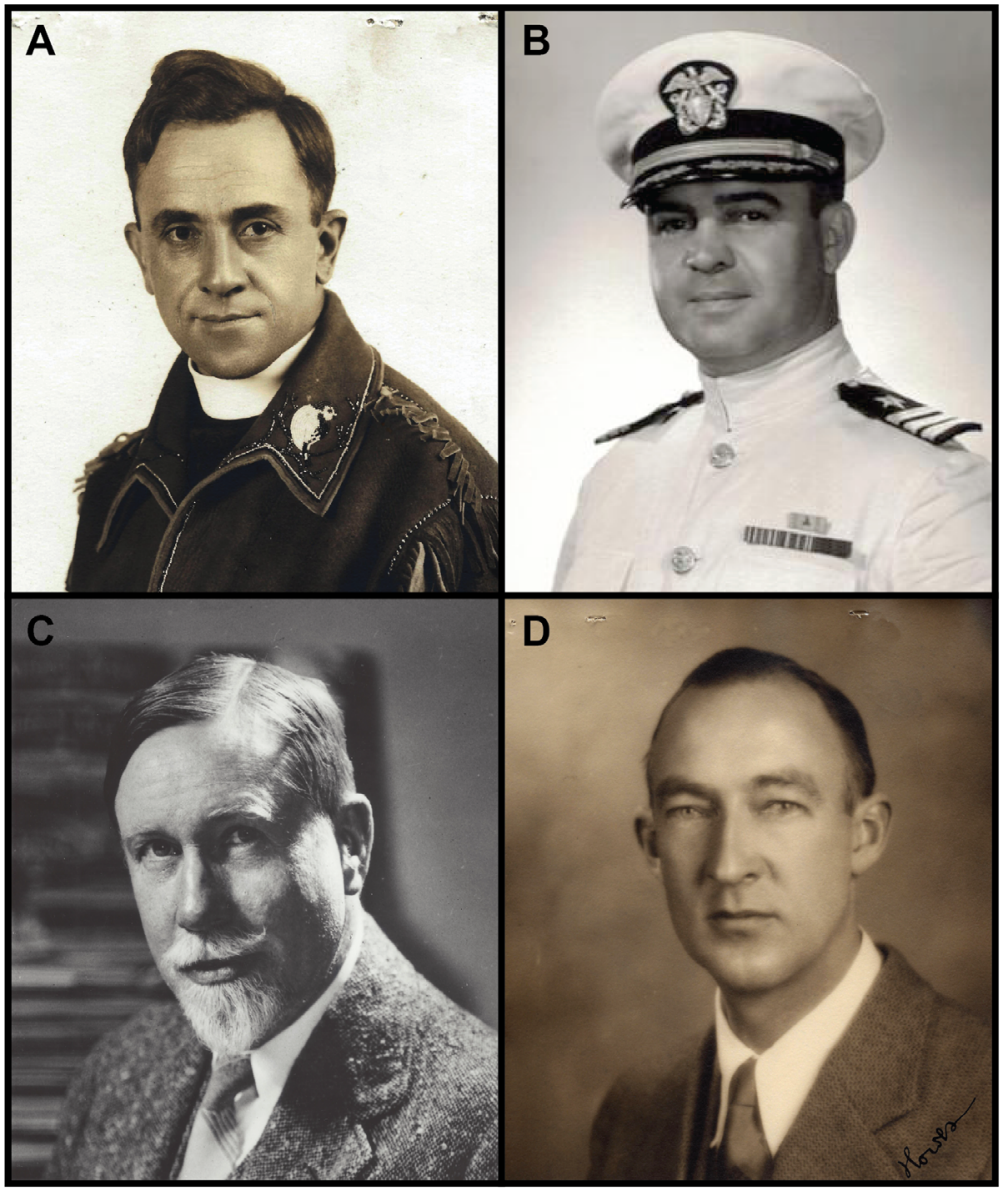
Explorers Club members discussed in the text. (A) Arctic explorer Father Bernard Rosecrans Hubbard, Society of Jesuits, University of Santa Clara. (B) Polar explorer Captain George Francis Kosco, United States Navy. (C) Circumnavigator, impresario, and ECAD Committee Chairman Commander Wendell Phillips Dodge. (D) Curator-Director of the Bruce Museum, Paul Griswold Howes. A, C, and D courtesy of The Explorers Club Research Collections. B courtesy of William G. Kosco.

One of the most famously strange scientific meals occurred on January 13, 1951, at the 47^th^ Explorers Club Annual Dinner (ECAD) when members purportedly dined on frozen woolly mammoth [11–13]. The prehistoric meat was supposedly found on Akutan Island in Alaska, USA, by the eminent polar explorers Father Bernard Rosecrans Hubbard (Fig 1A), “the Glacier Priest,” and Captain George Francis Kosco (Fig 1B) of the US Navy [11,14]. This much-publicized meal captured the public’s imagination and became an enduring legend and source of pride for the Club [8,11,13,15–17], popularizing an annual menu of “exotics” that continues today, making the Club as well-known for its notorious hors d’oeuvres like fried tarantulas and goat eyeballs as it is for its notable members such as Teddy Roosevelt and Neil Armstrong [18].

**Fig 2:**
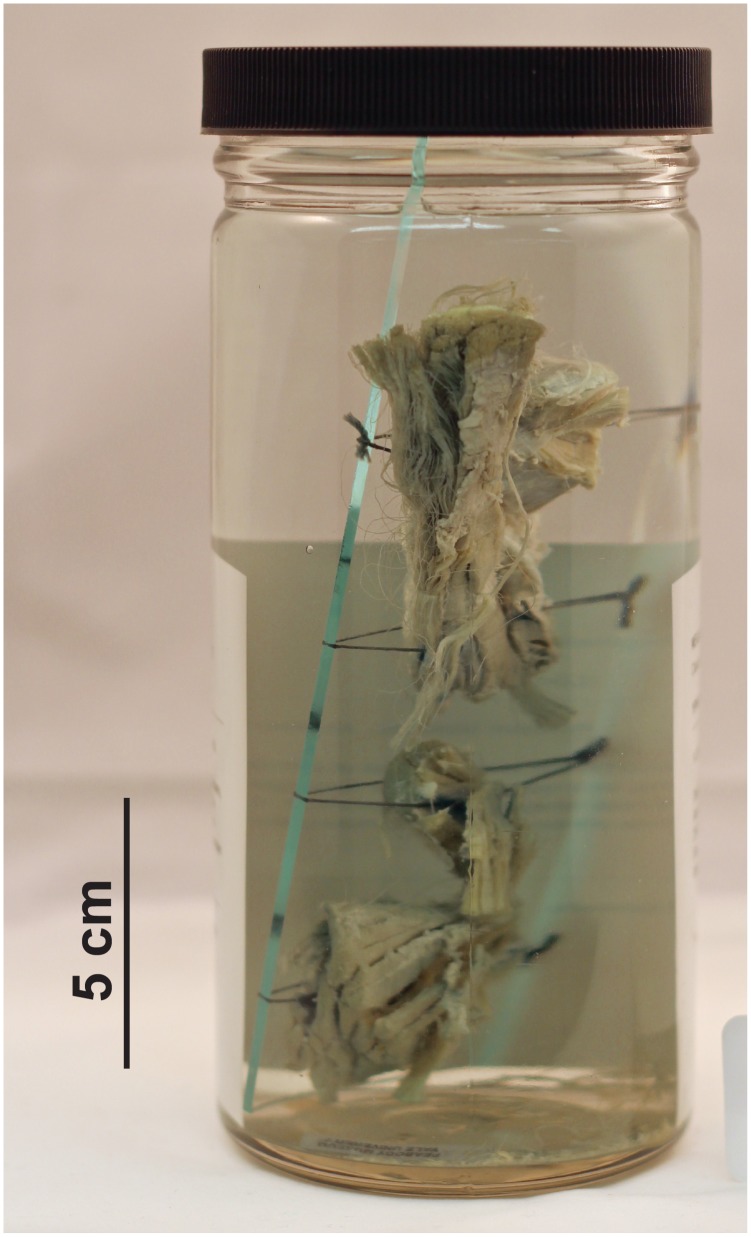
The cooked meat tissue served at the 1951 ECAD. Division of Vertebrate Zoology, YPM HERR 19475. Courtesy of the Peabody Museum of Natural History, Yale University, New Haven, CT, USA.

The Explorers Club mammoth might have remained an amusing, if not apocryphal, side note in the history of epicurism [8] except for two recently uncovered pieces of evidence. First, several small pieces of the meat (Fig 2) were preserved and placed in a museum, and second, the meat was never labeled as woolly mammoth, but was instead thought to be something much rarer, the extinct giant ground sloth *Megatherium* [14]. The meat was originally saved by the ECAD Committee Chairman and noted impresario, Commander Wendell Phillips Dodge (Fig 1C), and given to Paul Griswold Howes (Fig 1D) who was unable to attend the dinner and was eager to display a piece of the meal in the Bruce Museum (Greenwich, CT, USA) where he served as Curator-Director [14]. Dodge personally filled out the specimen label, confirming with Howes that the meat was *Megatherium*, not mammoth as an article in *The Christian Science Monitor* had reported [11,14].

**Fig 3:**
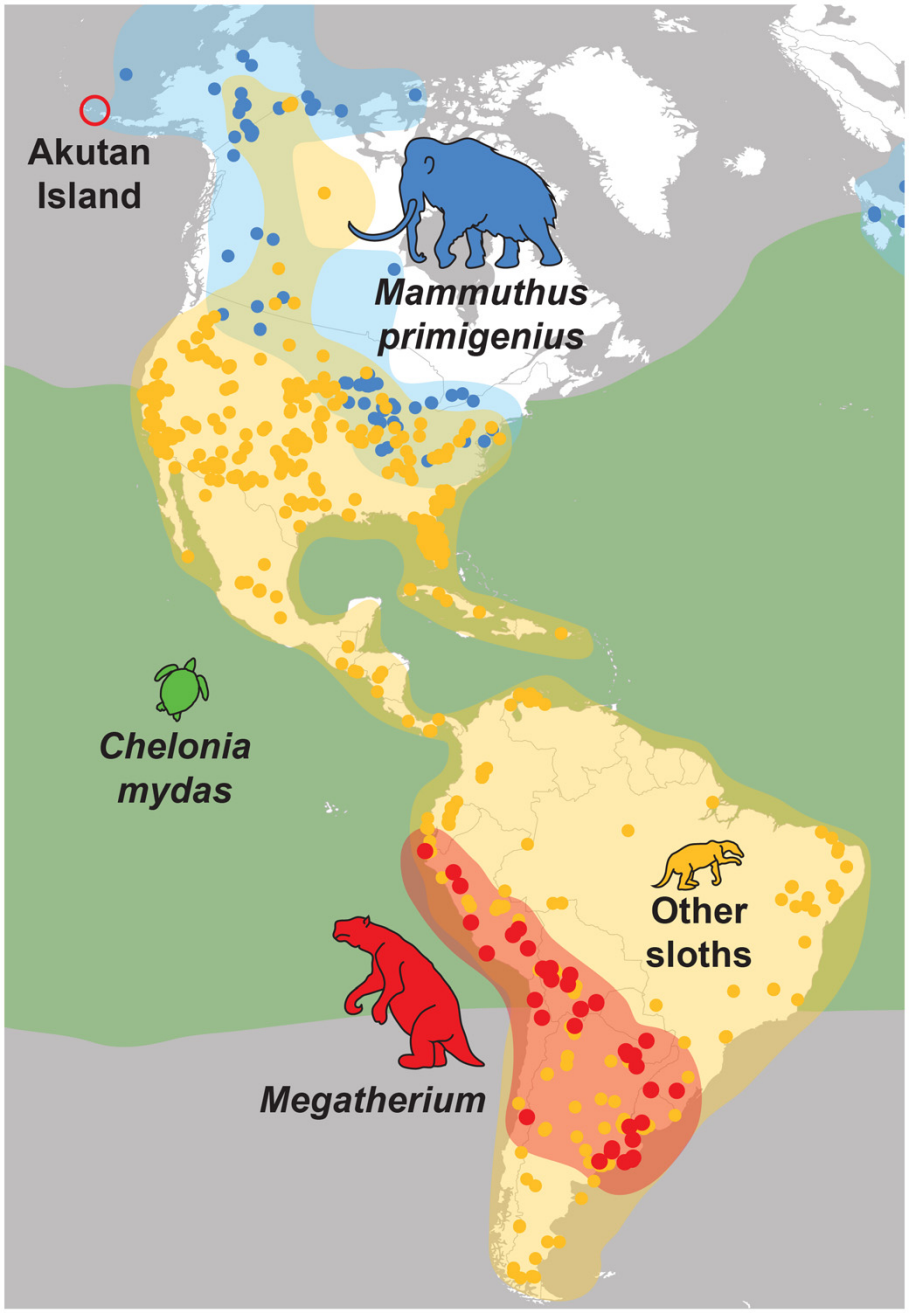
Geographic ranges of potential source taxa for the 1951 ECAD meat supposedly discovered on Akutan Island, AK, USA. Ranges are overlaid on a map of modern North and South America. Colored circles represent fossil localities: blue, woolly mammoth (*Mammuthus primigenius*); red, *Megatherium*; yellow, non-*Megatherium* fossil sloths. Green area represents modern range of green sea turtle (*Chelonia mydas*). Fossil specimen locality data were obtained from FAUNMAP II [51] and the Paleobiology Database (www.paleobiodb.org). The green sea turtle (*C*. *mydas*) range follows [25].

If the meat were in fact *Megatherium*, it would expand the latitudinal range of this genus, known only from South America, over 600% and rewrite what paleontologists know about ground sloth evolution (Fig 3) [19]. Even if the meat belonged to the distantly related North American ground sloth, *Megalonyx*, which lived as far north as central Alaska, it would still expand the known range of ground sloths by thousands of kilometers [20]. Although various researchers and explorers claim to have tasted extinct mammoth [6], mastodon [21], bison [7], and horse [22], we are unaware of any other attempt to serve ground sloth. There is also little direct evidence that ancient humans ever fed on ground sloths in North America [23,24]. Of the few sites that purport association of Paleoindians with ground sloths, almost all are South American [24]. Despite numerous fossil ground sloth remains in North America, only one individual bone is known with anthropogenic butcher marks [24], but due to this specimen’s uncertain collection history, the origin of these marks remains disputed [23]. If genuine, The Explorers Club meat would be the only conclusive example of humans, ancient or otherwise, consuming a North American ground sloth.

However, we found archival evidence that Dodge admitted the prehistoric meat was a hoax and misled Howes about its authenticity. In a circumlocutory editorial published soon after the dinner, Dodge fancifully described the sloth’s fossil history but hinted that he may have discovered “a potion by means of which he could change, say, *Cheylone mydas Cheuba* [*sic*] from the Indian Ocean into Giant Sloth from the ‘Pit of Hades’ in The Aleutians” [14]. Although endangered today [25], green sea turtle (*Chelonia mydas*) was once the preferred meat for turtle soup and was on the menu at the 1951 ECAD [14]. After building excitement by announcing an entrée of an unidentified extinct species, Dodge may have taken turtle meat meant for the evening’s soup and served it as *Megatherium* to the delight of members and the press [17]. The tongue-in-cheek style of Dodge’s “confession” only confused Explorers Club members, including Howes, who remained convinced the meat was ground sloth.

The potential scientific and historical importance of this specimen warranted investigation. Even if the meat were actually mammoth, or some other extinct mammal, the sample could still provide a valuable record of a previously unknown population, perhaps illuminating migration events across Beringia. Although no mammoth remains are known from Akutan Island, they have been reported on neighboring Unalaska Island, less than 20 km away [26], and on other volcanic Bering Sea isles like St. Paul [27]. Fossils on these isolated, volcanic islands initially perplexed researchers until it was shown that they all sat on a broad Beringian plain only exposed during glacials [27]. Megafauna could have easily walked to Akutan from mainland Siberia and Alaska and other islands like the Pribilofs. To determine the provenance and identity of the Explorers Club meat, we conducted an analysis of archival material relevant to the 1951 ECAD and used ancient DNA (aDNA) protocols to sequence the cooked muscle tissue.

## Materials and Methods

### DNA Extraction

The specimen of meat was originally designated BRCM 16925 before a transfer in 2001 from the Bruce Museum to the Yale Peabody Museum of Natural History (New Haven, CT, USA) where it gained the number YPM MAM 14399. The specimen is now permanently deposited in the Yale Peabody Museum with the designation YPM HERR 19475 and is accessible to outside researchers. The meat was never fixed in formalin and was initially stored in isopropyl alcohol before being transferred to ethanol when it arrived at the Peabody Museum. DNA extraction occurred at Yale University in a clean room with equipment reserved exclusively for aDNA analyses. We obtained five 250 mg samples of the cooked muscle tissue, which were air dried to remove all ethanol. We then mixed each sample with 3900 μL of EDTA digestion buffer (0.5 M, pH 8.0, UltraPure Invitrogen) and 100 μL of Proteinase K solution (20 mg/ml, Qiagen). Samples were incubated for 18 hours at 40°C, then for 12.5 hours at 61°C after an additional 40 μL of Proteinase K was added.

After digestion, as an initial purification step, samples were centrifuged for 3 min at 4000 rpm and the supernatant was transferred to a Centricon YM 30 (MWCO 30,000; Millipore) filter device. We then centrifuged samples at 4000 rpm for a minimum of 30 min to achieve a final volume of 250 μL. Next, we went through a second stage of purification using the MinElute PCR Purification Kit (Qiagen), following manufacturer’s guidelines and eluting 60 μl of dH_2_0. Due to the degraded nature of the DNA, we subsequently concentrated the five samples down to two by executing the MinElute protocol again (Qiagen), also eluting 60 μl of dH_2_0.

### Sequencing

We targeted a 308 base pair (bp) region of the cytochrome b (cytb) gene in the mitochondria using one pair of universal vertebrate primers from Kocher et al. [28]. The primers used were L14841 (5’-AAAAAGCTTCCATCCAACATCTCAGCATGATGAAA-3’) and H15149 (5’-AAACTGCAGCCCCTCAGAATGATATTTGTCCTCA-3’). PCR preparation was carried out in a laminar flow hood sterilized with UV light. DNA was amplified in 20 μL reactions consisting of Promega GoTaq *Thermus aquaticus* (*Taq*) DNA polymerase (ProMega) and a reaction buffer containing 1.5 mM MgCl_2_, 0.2 mM each dNTP, 0.25 mM forward and reverse primers, and 1 μg/ μL bovine serum albumin (BSA).

The PCR protocol consisted of 35 cycles of an initial denaturization period of 2 minutes at 95°C, followed by 35 cycles of denaturization at 95°C (1 min), annealing at 52°C (1 min), and extension at 72°C (1 min). This was followed by a final extension at 72°C (5 minutes) and a standby temperature of 4°C. The PCR was carried out in an Eppendorf Mastercycler DNA Thermocycler. In addition to the two concentrated “aDNA” samples, a blank reaction was included to monitor for contamination, as was a control sample of American angler (*Lophius americanus*), with which the primers were previously optimized. Amplified PCR products were run on a 2% agarose gel to confirm amplification success and purified with a polyethylene glycol precipitation protocol. All sequences were edited and aligned using Geneious version 8.0.4 [29].

### Identification

We used the BLAST nucleotide search function to match our DNA sequence with those registered in GenBank/EMBL and provide a preliminary identification. We calculated pairwise distances between the meat sample, 14 *Chelonia mydas* samples, *Mammuthus primigenius*, and *Nothrotheriops shastensis* (the closest ground sloth species to *Megatherium* with published cytb data) in MEGA v6.06 using the Maximum Composite Likelihood model (Table 1) [30,31]. For this analysis, we trimmed sequences to a 371 bp alignment.

**Table 1:** List of GenBank accession numbers for specimens used in phylogenetic and pairwise distance analyses.

Species	GenBank accession number	Locality	Original base pair length	Distance from YPM HERR 19475
YPM HERR 19475	KT276329	Explorers Club Annual Dinner, 1951 New York, NY, USA	363	NA
*Nothrotheriops shastensis*	AF232015	Nevada, USA	648	0.341
*Mammuthus primigenius*	DQ316067	Russia	369	0.364
*Chelonia mydas*	EU918367	South China Sea	1144	0.031
*Chelonia mydas*	EU918368	South China Sea	1144	0.031
*Chelonia mydas*	JX454971	Hawaii, USA	1144	0.034
*Chelonia mydas*	JX454974	Archipiélago de Revillagigedo, Mexico	1144	0.034
*Chelonia mydas*	JX454978	Galapagos Islands, Ecuador	1144	0.034
*Chelonia mydas*	JX454976	Sipadan, Malaysia	1144	0.028
*Chelonia mydas*	JX454985	Yap, Micronesia	1144	0.028
*Chelonia mydas*	EU787021	Zapatilla, Panama	1070	0.017
*Chelonia mydas*	JQ026233	Costa Rica	1144	0.017
*Chelonia mydas*	JX454990	Tortugero, Costa Rica	1144	0.017
*Chelonia mydas*	JQ034420	Suriname	1144	0.017
*Chelonia mydas*	JX454972	Karpaz, Cyprus	1144	0.017
*Chelonia mydas*	AB012104	Atlantic Ocean	1144	0.017
*Chelonia mydas*	NC000886	Isla Mujeres, Mexico	1144	0.017
*Caretta caretta*	KP256531	Colombia	1144	0.117
*Eretmochelys imbricata*	KP221806	Colombia	1144	0.109

Pairwise genetic distances between specimens and the YPM HERR 19475 sample were calculated in MEGA v.6.06 using the Maximum Composite Likelihood Model [30,31].

**Fig 4:**
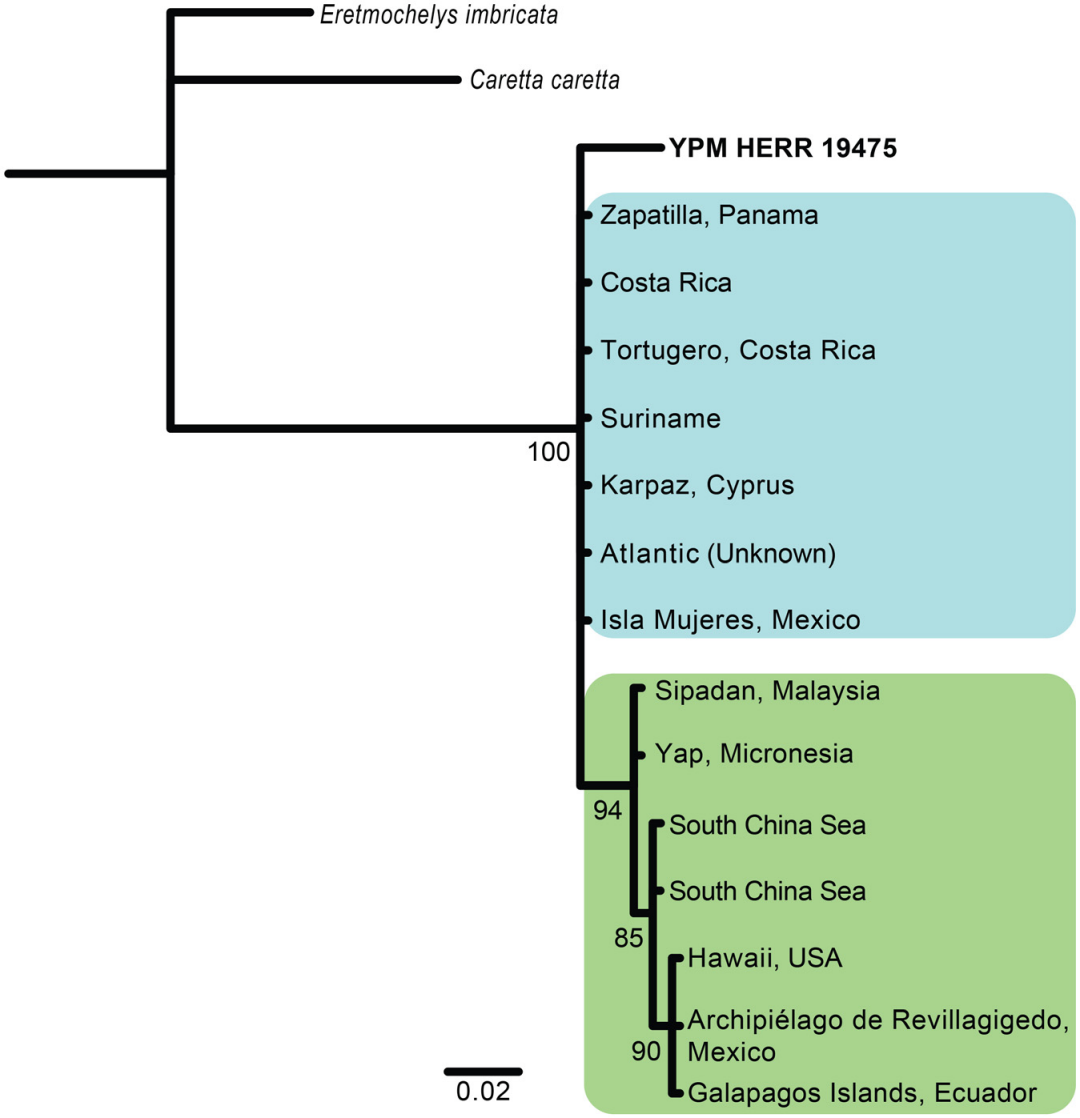
Phylogenetic tree of *C*. *mydas* and relatives. Phylogeny based on a 369 bp alignment of the mitochondrial cytochrome-b gene, inferred from Bayesian analyses. Specimens from the Pacific Ocean form a clade (green), whereas those with Atlantic or Mediterranean origins (blue) are unresolved. The YPM specimen is bolded. Node labels indicate posterior probability percentage support estimates. All branch lengths are in substitution units.

To determine the geographic origin of the specimen, we produced a phylogeny of *Chelonia mydas* using cytb sequences available on GenBank. Fourteen sequences with locality information were available on GenBank and were trimmed to a 369 bp alignment (Fig 4, Table 1). Bayesian analyses were used to estimate the phylogenetic tree, executed in MrBayes version 3.2.5 [32,33]. *C*. *mydas* cytb samples were analyzed as a single partition (1 million generations, nruns = 2, nchains = 4, burninfrac = .25) using the molecular evolutionary model HKY. We used Markov chain Monte Carlo methods to approximate posterior probabilities and parameter values, and used an invariant gamma rates unconstrained model with a rate parameter of 10.0. We monitored the average SD of the split frequencies between the two runs to assure convergence of the MrBayes runs, ensuring the SD value was < 0.01. The hawksbill sea turtle (*Eretmochelys imbricata*) and the loggerhead sea turtle (*Caretta caretta*), both in the family Cheloniidae, were used as outgroups to root the tree (Table 1).

## Results

We successfully amplified the cytb gene from the cooked muscle tissue. The two samples yielded identical sequences that were 363 bp long after editing (Genbank Accession No. KT276329, Table 1). A BLAST search produced 14 close matches belonging to *Chelonia mydas*, with low pairwise distance values ranging from 0.017 to 0.034 (Table 1). The mammoth *M*. *primigenius* and the ground sloth *N*. *shastensis* had much greater genetic distance values (0.364 and 0.341, respectively) (Table 1). We were unable to resolve the geographic origin of the YPM specimen with a phylogenetic analysis including the 14 other cytb fragments from Genbank belonging to *C*. *mydas* specimens with known geographic information (Fig 4, Table 1).

## Discussion

The genetic data indicate that the meat served at the 1951 ECAD was not prehistoric, but sea turtle, likely from the soup served during the same meal. We acknowledge it is possible that members of the Club actually consumed *Megatherium* or mammoth meat but that Dodge sent Howes a sample from the wrong dish; however, this seems unlikely. There is no archival evidence suggesting that Hubbard or Kosco discovered a frozen mammoth or ground sloth. Hubbard described other specimens he found in Alaska [34] but out of the hundreds of photographs he took while exploring Akutan and Unimak (a neighboring island sometimes listed as the *Megatherium* specimen’s locality), none feature anything resembling a carcass of a mammoth or ground sloth (D. Dominguez, pers. comm.). Kosco frequently explored uninhabited islands in the Pacific and often shared interesting discoveries of his voyages with his family or other members of The Explorers Club (W.G. Kosco, pers. comm.) [35]. However, we were unable to find any instance where he described frozen mammoth or ground sloth.

Curiously, Arnold Hauerslev Haverlee, Club member and chef for several Explorers Club dinners, claimed he did cook mammoth meat at the 1951 ECAD [36]. When the Sportsmen’s Club of South Glastonbury, CT, USA, publicly contacted The Explorers Club in 1957 for assistance in finding mammoth for their own annual dinner, Haverlee guaranteed the sportsmen that for a $20,000 fee, he could find and cook mammoth meat [36]. Haverlee was nationally famous for his role as chef at the extravagant 50^th^ ECAD in 1954 where he served oddities such as polar bear and fried termites to honorary guests Tenzing Norgay and Werner non Braun [37], but we could find no independent evidence that he was also the 1951 ECAD mammoth chef. Had he actually cooked prehistoric meat, it likely would have been mentioned in his biography [38] or in the *Explorers Cookbook* where he described recipes for his other exotic dishes [39]. Fellow Explorers Club member Coleman Shaler Williams provided a detailed recipe in the book for the fossil horse he claimed to have served at the 1969 ECAD (See S1 Appendix for the authenticity of this horse dish) [22]. It is likely that Haverlee’s description of cooking mammoth meat was all in jest, playing along with the Sportsmen’s Club tongue-in-cheek publicity stunt. The previous year, the South Glastonbury Sportsmen lamented that they had been unable to bag a pterodactyl for the planned main course [40].

Our archival research suggests that the prehistoric meat served at the 1951 ECAD was a jocular publicity stunt that mistakenly wound its way into fact [12,13]. Although there was one previous incident of a paleontologist mistaking a ground sloth for a sea turtle [41], it still seems odd that a skilled naturalist like Howes, as well as other Explorers Club members and journalists, continued to believe in the authenticity of the sloth meat even after Dodge admitted it was a playful prank. However, Dodge’s facetious admission of guilt [14], published in the Club’s *Explorers Journal*, was confusing to Club members and later researchers, and may not have been taken for the confession that it was [8,12,13]. The designation of Hubbard and Kosco as the collectors also gave the story an air of authenticity. Hubbard, a Jesuit priest, taught in the Geology Department at the University of Santa Clara and was famous for his expeditions to the volcanoes and ice fields of Alaska [42]. Kosco led several polar expeditions, earned an eponymous glacier in Antarctica, and collected specimens for the Smithsonian [43,44]. At the time of the 1951 ECAD, Kosco was living on Kodiak Island, Alaska, and arranged for the U.S. Navy to fly king crabs, table settings of arctic vegetation, and glacial ice (for chilling cocktails) back to New York for the dinner at the Roosevelt Hotel (W.G. Kosco, pers. comm.) [14]. Two well-known polar explorers finding a frozen mammoth or sloth on an island similar to those where fossil mammoths had already been unearthed must not have seemed extraordinary, especially for a Club that keeps a mutant, four tusked elephant in its trophy room and whose members discovered the North and South Poles.

Although it remains unclear why later accounts identify the 1951 ECAD meat almost exclusively as mammoth instead of sloth [8,12,15], one Club member, Lieutenant Colonel Herbert Bishop Nichols, may bear some responsibility. Nichols, the first science editor for *The Christian Science Monitor* [45], published the most detailed, and likely influential, description of the meat four days after the 1951 ECAD and claimed it was mammoth [11]. It is unknown why he chose “mammoth” when other members explicitly told reporters they had eaten “sloth” [16,46] but the article is taken as proof by modern journalists that mammoth was actually served at the dinner [12,13].

Tall tales like The Explorers Club “mammoth” can have a lasting impact on scientific thought and the public’s perception of natural history. A fictitious account of one frozen carcass written for a German children’s book in 1859 is cited heavily in early scientific literature and is still reproduced as fact in some paleontological textbooks today [47,48]. A story in *McClure’s Magazine* recounting the hunting of a live mammoth later donated to the Smithsonian proved so popular that the museum had to issue a public statement denying the specimen’s existence and challenging donors to fund an expedition to seek out a real frozen mammoth with which to replace it [49].

More importantly, historical museum metadata such as collection localities are frequently aggregated and used as the basis for macroecological studies, but these data are susceptible to numerous, significant sources of error from careless labeling to purposeful fraud, and are rarely validated before downstream analyses [50]. The responsibility of maintaining accurate specimen records falls to the museum housing those specimens; however, our findings further emphasize the value of collecting and curating voucher specimens, even those with contentious metadata, as evidence for extraordinary claims. Presaging this study, Dodge ended his *mea culpa* about the mystery meat by writing, “Science in all its Divisions must form the Jury and decide the fate of the, shall we say, ‘Defendant’.”[14]. Had Howes not accessioned the “ground sloth” into his museum, the identity of the “Defendant” could never have been examined. Sending Howes his sample years before the structure of DNA had been discovered, Dodge probably never anticipated that one day the several nondescript pieces of meat he saved would finally lay to rest the myth of The Explorers Club “mammoth.”

## Supporting Information

S1 Appendix The authenticity of the fossil horse purportedly served at the 1969 ECAD. (DOCX)
