# Supplementary material for: Was Frozen Mammoth or Giant Ground Sloth Served for Dinner at The Explorers Club?
Source: PLoS One. 2016 Feb 3;11(2):e0146825. doi: 10.1371/journal.pone.0146825 (PMC4740485; doi:10.1371/journal.pone.0146825)
Supplement: S1 Appendix — (DOCX) [file pone.0146825.s001.docx]

**S1 Appendix. The authenticity of the fossil horse purportedly served at the 1969 ECAD.**

In addition to frozen ground sloth (*Megatherium*) or woolly mammoth (*Mammuthus primigenius*), the menu of the Explorers Club Annual Dinner (ECAD) has also featured fossil horse. Coleman “Coley” Shaler Williams was a Club member and longtime field assistant for the influential paleontologist George Gaylord Simpson at the American Museum of Natural History (AMNH). In 1969, Williams presented a dish at the 65^th^ ECAD supposedly made from bone marrow of a well preserved extinct horse (*Equus alaskensis*) [1,2] left over from radiocarbon sampling by Professor Russell Dale Guthrie of the University of Alaska, Fairbanks, USA (R. D. Guthrie, pers. comm.). We were unable to find a specimen matching Williams’ exact description despite examining thousands of horse fossils housed in the Vertebrate Paleontology collections of the AMNH. However, Guthrie's later consumption of a piece of extinct steppe bison (*Bison priscus*) found frozen in permafrost [3] lends credibility to the ancient origin of the 1969 dinner of "paleoequine marrow balls."

**References**

1. Williams CS. Coleman S. Williams. In: Douglas LA, Douglas CE, editors. The Explorers Cookbook. Caldwell: Caxton Printers; 1971. pp. 219–220.

2. Yummy: reindeer eyeballs. The Hartford Courant. 12 Jul 1969. p. 9.

3. Guthrie RD. Frozen Fauna of the Mammoth Steppe: The Story of Blue Babe. Chicago: The University of Chicago Press; 1990.
